# Determinants of First-Trimester Residual Myometrial Thickness After Previous Cesarean Delivery: A Retrospective Cohort Study

**DOI:** 10.3390/medicina62071384

**Published:** 2026-07-17

**Authors:** Coşkun Orhaner, Bilge Çetinkaya Demir

**Affiliations:** 1 Department of Gynecology and Obstetrics, Soranus IVF Center, 16070 Bursa, Turkey; 2 Department of Gynecology and Obstetrics, Faculty of Medicine, Uludağ University, 16120 Bursa, Turkey; bilgecd@uludag.edu.tr

**Keywords:** cesarean section, cesarean scar defect, residual myometrial thickness, transvaginal ultrasonography, pregnancy outcomes

## Abstract

*Background and Objectives:* To investigate factors associated with first-trimester residual myometrial thickness (RMT) at the site of a previous cesarean section scar and to evaluate the relationship between RMT and selected pregnancy and neonatal outcomes. *Materials and Methods:* This retrospective cohort study included 80 pregnant women with at least one previous CS who attended the Department of Obstetrics and Gynecology, Uludağ University Faculty of Medicine, between July 2017 and December 2018. RMT at the cesarean scar site was measured by transvaginal ultrasonography (TVUS) during the first trimester. After exclusion of 15 women because of incomplete records or missing follow-up data, 65 patients were included in the final analysis. Participants were categorized according to first-trimester RMT as a thin scar group (RMT ≤ 8 mm, n = 37) and a control group (RMT > 8 mm, n = 28). Demographic characteristics, obstetric history, cesarean-related variables, postpartum hemorrhage (PPH) history, ultrasonographic findings, and neonatal outcomes were analyzed. Statistical analyses included Student’s *t*-test, chi-square test, one-way analysis of variance (ANOVA), and Spearman correlation analysis. *Results:* Women with thinner residual myometrium had significantly higher parity (*p* = 0.003), a greater number of previous cesarean deliveries (*p* = 0.016), and a higher frequency of previous PPH (*p* = 0.037) compared with controls. Mean RMT was significantly lower in women with parity ≥ 2 than in women with parity 1 (6.39 ± 2.76 mm vs. 8.51 ± 2.81 mm, *p* = 0.003). Similarly, women with a history of PPH demonstrated significantly lower RMT than those without such a history (5.67 ± 2.11 mm vs. 7.82 ± 2.98 mm, *p* = 0.037). Correlation analysis revealed a moderate inverse relationship between the number of previous cesarean deliveries and RMT (r = −0.463, *p* < 0.001). No significant group differences were observed regarding cervical length, placental location, gestational age at delivery, 5 min Apgar score, or birth weight. *Conclusions:* First-trimester RMT was significantly associated with parity, previous cesarean delivery number, and a history of PPH. Among the evaluated variables, the number of previous cesarean deliveries demonstrated the strongest independent association with reduced RMT. These findings suggest that early transvaginal ultrasonographic assessment of RMT may serve as a useful sonographic marker of cesarean scar morphology and healing characteristics in pregnancies following cesarean delivery. Larger prospective studies are needed to determine the clinical significance of first-trimester RMT measurements for subsequent obstetric outcomes.

## 1. Introduction

Cesarean section is one of the most commonly performed surgical procedures worldwide, and its frequency has increased substantially over recent decades. Although cesarean delivery is often a life-saving intervention for both mother and fetus, the growing number of women with previous cesarean deliveries has led to increasing concern regarding long-term complications associated with uterine scar healing. Incomplete healing of the uterine incision may result in the formation of a cesarean scar defect (CSD), also known as a niche or isthmocele, which has been associated with abnormal uterine bleeding, chronic pelvic pain, infertility, cesarean scar pregnancy, placenta accreta spectrum disorders, uterine rupture, and difficulties during subsequent gynecological procedures. The reported prevalence of clinically relevant scar defects varies widely, ranging from approximately 19% to 88%, depending on the study population and diagnostic method used [1].

Ultrasonographic studies have demonstrated that CSDs are common findings after cesarean delivery. In a landmark study involving women examined 6–9 months after delivery, Osser et al. reported scar defects in 61% of women after one CS, 81% after two CSs, and 100% after three or more CSs. Furthermore, myometrial thickness progressively decreased from 8.3 mm after one CS to 4.7 mm after three or more CSs, suggesting cumulative deterioration of scar integrity with repeated uterine incisions [2]. Similar findings were reported by Wang et al., who demonstrated that women with multiple previous cesarean deliveries had significantly larger and deeper scar defects than women with a single previous CS [3]. Likewise, Ofili-Yebovi et al. identified deficient scars in 19.4% of women and showed that multiple cesarean deliveries were independently associated with scar deficiency [4]. These findings have subsequently been confirmed by a systematic review demonstrating that multiple previous cesarean deliveries represent one of the most consistent risk factors for niche formation and incomplete scar healing [5].

Although repeated cesarean delivery appears to be a major determinant of scar morphology, several additional factors have been implicated in impaired uterine healing. Vikhareva Osser and Valentin demonstrated that cesarean delivery performed during advanced labor was strongly associated with large scar defects, with increasing cervical dilatation, prolonged labor, low fetal station, and oxytocin augmentation all significantly increasing the risk of incomplete scar healing [6]. In a prospective cohort study, Antila-Långsjö et al. identified previous cesarean delivery, increased maternal body mass index, and gestational diabetes mellitus as independent risk factors for isthmocele formation, while prolonged active labor further increased the risk among women undergoing emergency cesarean delivery [7]. Furthermore, Pomorski et al. reported that RMT decreases with increasing numbers of cesarean deliveries and is negatively associated with uterine retroflexion, supporting the concept that scar healing is influenced by both obstetric and anatomical factors [8].

Accurate assessment of cesarean scar morphology has therefore become increasingly important. TVUS is currently the most widely used modality for evaluation of cesarean scars because of its accessibility, reproducibility, and noninvasive nature. However, considerable heterogeneity has historically existed regarding scar definitions and measurement techniques. To address this issue, Naji et al. proposed a standardized ultrasonographic approach for describing cesarean scars and recommended uniform assessment of scar dimensions and RMT [9]. More recently, an international consensus process conducted by the European Niche Taskforce established detailed recommendations for sonographic niche evaluation and identified RMT, adjacent myometrial thickness, niche depth, niche length, and niche width as essential parameters that should be reported in future studies [10]. These efforts have substantially improved the comparability of ultrasonographic investigations evaluating cesarean scar healing.

Among the various sonographic parameters used to characterize cesarean scars, RMT has emerged as one of the most clinically relevant measurements. Reduced RMT reflects thinning of the myometrium overlying the scar and may indicate impaired structural integrity of the lower uterine segment. The clinical significance of scar morphology has been demonstrated in several studies. Bij de Vaate et al. reported that women with a niche experienced significantly more postmenstrual spotting than women without a niche and showed that larger niche volumes were associated with more severe symptoms [11]. In their systematic review, Bij de Vaate et al. further concluded that postmenstrual spotting was the symptom most consistently associated with CSDs [5]. Similarly, Au et al. demonstrated that women with ultrasonographically detectable scar defects had significantly higher rates of failed medical termination of pregnancy and that outcomes worsened as RMT decreased relative to adjacent myometrium, emphasizing the clinical relevance of scar measurements [12].

Most available evidence regarding cesarean scar morphology originates from studies conducted in non-pregnant women. Nevertheless, increasing attention has been directed toward scar assessment during pregnancy, particularly in the first trimester. Stirnemann et al. demonstrated that cesarean scars can be visualized reliably during first-trimester TVUS, with successful identification in more than 99% of examined women, suggesting that early pregnancy assessment may facilitate recognition of patients at risk for subsequent complications [13]. Baranov et al. subsequently showed that the sonographic appearance of cesarean hysterotomy scars at 11–14 weeks closely resembles that observed before pregnancy, supporting the reliability of first-trimester scar evaluation [14]. Furthermore, a prospective longitudinal study by Naji et al. demonstrated that scar dimensions continue to change throughout pregnancy and that RMT progressively decreases as gestation advances, indicating that early pregnancy measurements may provide important baseline information regarding subsequent scar remodeling [15].

Despite substantial advances in the understanding of CSDs, important knowledge gaps remain. While previous studies have focused primarily on scar prevalence, niche morphology, surgical factors, and reproductive outcomes, relatively limited information is available regarding the relationship between first-trimester RMT and maternal obstetric characteristics. In particular, the influence of parity, number of previous cesarean deliveries, PPH history, and other clinical variables on early pregnancy scar morphology remains insufficiently characterized. Clarification of these associations may improve identification of women at increased risk for impaired scar healing and future scar-related complications.

Therefore, the aim of the present study was to evaluate RMT during the first trimester of pregnancy in women with a history of cesarean delivery and to investigate its association with demographic characteristics, obstetric history, ultrasonographic findings, and selected pregnancy and neonatal outcomes. In addition, we sought to identify clinical factors associated with reduced RMT, with particular emphasis on parity, previous cesarean delivery number, and PPH history. Because most previous studies have focused on non-pregnant women, late-pregnancy assessments, or detailed niche characterization, data regarding factors associated with first-trimester RMT remain limited. Accordingly, the present study aimed to provide additional evidence regarding early-pregnancy cesarean scar morphology and its relationship with maternal, obstetric, and perinatal characteristics.

## 2. Materials and Methods

### 2.1. Study Design and Participants

This retrospective cohort study was conducted at the Department of Obstetrics and Gynecology, Bursa Uludağ University Faculty of Medicine, Türkiye. Ethical approval was obtained from the Clinical Research Ethics Committee of Bursa Uludağ University Faculty of Medicine (Approval No: 2017-12/2), and all procedures were performed in accordance with the principles of the Declaration of Helsinki.

The study population consisted of pregnant women with a history of at least one previous cesarean delivery who attended the antenatal outpatient clinic between 20 July 2017 and 31 December 2018 and underwent routine first-trimester ultrasonographic examination. Medical records, ultrasonographic findings, pregnancy follow-up data, and delivery outcomes were reviewed retrospectively.

The inclusion criteria were: (1) maternal age ≥ 18 years, (2) viable singleton intrauterine pregnancy, (3) ultrasonographic examination performed between 11 and 14 weeks of gestation, (4) history of previous cesarean delivery through a lower uterine segment transverse incision, and (5) absence of known uterine anomalies. Women with a history of classical CS, previous uterine surgery other than cesarean delivery, multiple pregnancy, or incomplete clinical and ultrasonographic records were excluded.

**Figure 1:**
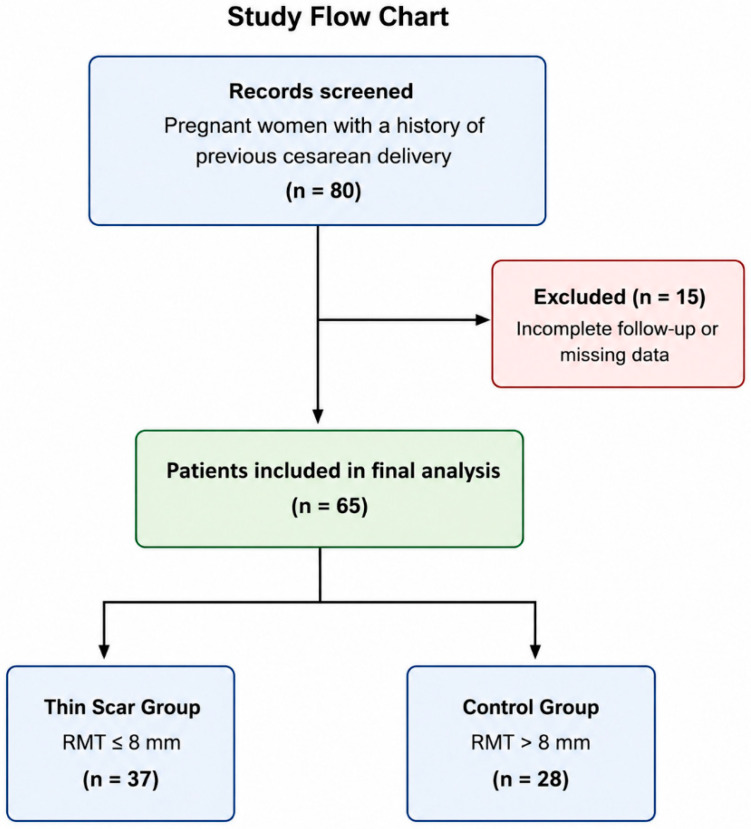
Study flow diagram illustrating patient selection and group allocation. Eighty pregnant women with a history of previous cesarean delivery were screened. After exclusion of 15 cases because of incomplete records or missing follow-up data, 65 women were included in the final analysis. Patients were categorized according to first-trimester RMT measured by TVUS into a thin scar group (RMT ≤ 8 mm; n = 37) and a control group (RMT > 8 mm; n = 28).

Initially, 80 women with a history of previous cesarean delivery were identified. Fifteen patients were excluded because of incomplete follow-up data (n = 4), missing ultrasonographic measurements (n = 3), unavailable delivery records (n = 5), or insufficient clinical data (n = 3). Consequently, 65 women fulfilled the study criteria and were included in the final analysis. Only women with complete pregnancy follow-up data and available delivery outcomes were included in the final analysis (Figure 1). Cases with missing outcome information or unavailable delivery records were excluded to minimize outcome ascertainment bias.

The primary objective of the study was to investigate factors associated with RMT at the site of a previous cesarean scar during the first trimester of pregnancy and to evaluate the relationship between scar thickness and subsequent obstetric outcomes.

### 2.2. Clinical Assessment and Data Collection

Maternal demographic, obstetric, ultrasonographic, and neonatal data were obtained from institutional electronic databases and archived patient records. The collected demographic and obstetric characteristics included maternal age, gravidity, parity, number of abortions, number of previous cesarean deliveries, interval since the previous CS, gestational age at previous cesarean delivery, mode of previous CS (elective or emergency), and history of PPH. Information regarding PPH was extracted from the medical records as a binary historical variable (present/absent). Detailed information regarding the etiology, severity, estimated blood loss, transfusion requirement, and the specific pregnancy in which PPH occurred was not consistently available in the retrospective records.

Pregnancy-related variables included cervical length measured during first-trimester ultrasonography, placental localization, antenatal vaginal bleeding, and gestational age at delivery. Neonatal outcomes included birth weight and first- and fifth-minute Apgar scores. All data were reviewed and verified before statistical analysis to ensure consistency and completeness. All included deliveries occurred at Bursa Uludağ University Faculty of Medicine, and delivery outcomes were obtained from the institutional electronic medical record system. This approach enabled complete ascertainment of delivery and neonatal outcome data for all patients included in the final analysis.

### 2.3. Ultrasonographic Evaluation of the Cesarean Scar

All participants underwent first-trimester transvaginal ultrasonographic examination as part of routine antenatal assessment. Ultrasonographic evaluation was performed using a PHILIPS ClearVue 550 ultrasound system equipped with a 4–9 MHz transvaginal probe. Examinations were carried out with the patient in the lithotomy position and with an empty urinary bladder. All measurements were performed by the study investigator under the supervision of the thesis advisor, who was experienced in obstetric ultrasonography.

For optimal visualization of the lower uterine segment, the ultrasound depth was adjusted to obtain a panoramic midsagittal view of the uterine cavity. Following identification of the previous cesarean scar, magnification was increased so that the lower uterine segment and scar region occupied approximately 75% of the ultrasound screen. The endocervical canal, internal cervical os, external cervical os, lower uterine segment, urinary bladder, and uterovesical fold were identified before measurements were obtained. Care was taken to avoid excessive pressure on the cervix during image acquisition because probe-induced compression may influence cervical and lower uterine segment measurements.

**Figure 2:**
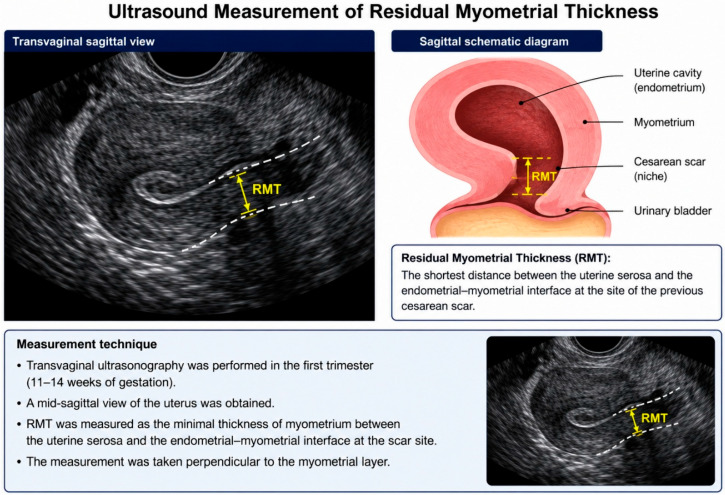
Ultrasound measurement of RMT at the site of a previous cesarean scar. TVUS was performed during the first trimester in a midsagittal uterine plane. RMT was defined as the shortest distance between the uterine serosa and the endometrial–myometrial interface at the cesarean scar site. The schematic illustration demonstrates the anatomical relationship between the cesarean scar niche, myometrium, uterine cavity, and urinary bladder, together with the measurement technique used for RMT assessment.

The cesarean scar was examined morphologically in the midsagittal plane. RMT was defined as the shortest measurable distance between the uterine serosa and the endometrial–myometrial interface at the site of the previous cesarean scar. Measurements were obtained at the thinnest portion of the residual myometrium and perpendicular to the myometrial layer to minimize measurement error. The measurement technique used in the present study is illustrated in Figure 2.

For scar identification, the lower uterine segment was systematically examined in the midsagittal plane. When present, a cesarean scar niche was identified as an anechoic indentation at the site of the previous cesarean scar visible on transvaginal ultrasonography. In cases of suboptimal visualization, image acquisition was repeated after adjustment of probe position, magnification, and insonation angle to optimize delineation of the scar margins and residual myometrium. Measurements were recorded only when the scar region and residual myometrial layer could be clearly visualized according to the standardized imaging protocol used throughout the study.

To assess measurement reproducibility, RMT measurements were repeated in 20 randomly selected examinations by the same observer. Intraobserver agreement was evaluated using the intraclass correlation coefficient (ICC). The reproducibility analysis demonstrated excellent agreement (ICC = 0.92, 95% CI: 0.86–0.96), indicating high reliability of the RMT measurements.

When present, cesarean scar niches (isthmoceles) were documented during ultrasonographic examination as part of the routine scar assessment. However, the primary objective of the present study was the evaluation of RMT and its association with maternal, obstetric, and neonatal outcomes. Therefore, statistical analyses were focused on RMT measurements. Cervical length was measured during the same examination using standard transvaginal ultrasonographic techniques. Although cesarean scar niches were documented when identified during ultrasonographic examination, detailed niche characterization parameters such as niche depth, width, length, adjacent myometrial thickness, and residual-to-adjacent myometrial thickness ratio were not consistently available in all archived examinations. Therefore, the present study focused on RMT as the primary and most consistently recorded sonographic parameter of scar morphology across the study cohort.

### 2.4. Study Groups and Outcome Measures

To investigate factors associated with scar thickness, participants were categorized according to first-trimester RMT measurements. Women with an RMT of 8 mm or less were classified as the Thin Scar Group (n = 37), whereas women with an RMT greater than 8 mm were assigned to the Control Group (n = 28). Because no universally accepted first-trimester RMT cutoff has been established for the assessment of cesarean scar integrity during pregnancy, the 8 mm threshold was selected as a pragmatic study-specific grouping criterion. This threshold approximated the central tendency of RMT values observed in the study cohort and allowed clinically interpretable comparisons between women with relatively thinner and thicker residual myometrium while maintaining balanced group sizes. This threshold was used solely to facilitate comparative analyses between women with relatively thinner and thicker residual myometrium and should not be interpreted as a validated clinical cutoff for predicting adverse obstetric outcomes. Because no validated first-trimester RMT threshold currently exists, the dichotomization was performed solely for exploratory comparative analyses, and all major findings were additionally evaluated using continuous-variable correlation and multivariable regression analyses. Furthermore, RMT was analyzed as a continuous variable to minimize the limitations associated with dichotomization, including potential information loss and misclassification. Therefore, the primary interpretation of the study is based on continuous-variable analyses, whereas comparisons using the 8 mm threshold should be considered exploratory and descriptive rather than indicative of a validated clinical cutoff.

The primary outcome measure was RMT measured at the site of the previous cesarean scar. Secondary outcome measures included the evaluation of associations between RMT and maternal age, parity, number of previous cesarean deliveries, history of PPH, cervical length, placental localization, gestational age at delivery, neonatal birth weight, and Apgar scores. In addition, the relationship between the number of previous cesarean deliveries and RMT was assessed using correlation analysis.

### 2.5. Statistical Analysis

Statistical analyses were performed using IBM SPSS Statistics software version 26.0 (IBM Corp., Armonk, NY, USA). Continuous variables were expressed as mean ± standard deviation (SD), whereas categorical variables were presented as frequencies and percentages.

The distribution of continuous variables was assessed using visual inspection of histograms and probability plots together with the Shapiro–Wilk test. Comparisons between the Thin Scar Group (RMT ≤ 8 mm) and the Control Group (RMT > 8 mm) were performed using the independent-samples Student’s *t*-test for continuous variables. Categorical variables were compared using the Pearson chi-square test or Fisher’s exact test, as appropriate.

To evaluate the relationship between RMT and clinical variables, correlation analyses were performed using Spearman’s rank correlation coefficient (ρ). Correlation strength was interpreted according to conventional criteria as weak (|r| < 0.30), moderate (0.30–0.49), or strong (≥0.50).

Differences in RMT according to gravidity category, parity, abortion history, mode of previous cesarean delivery, placental localization, PPH history, and antenatal vaginal bleeding were evaluated using independent-samples Student’s *t*-test for binary variables and ANOVA for variables with more than two categories, as appropriate.

To identify factors independently associated with RMT, a multivariable linear regression analysis was performed. Variables considered clinically relevant or showing an association with RMT in univariable analyses were entered into the regression model. Regression coefficients (β), 95% confidence intervals (CIs), and *p*-values were reported.

Descriptive boxplots were generated to visualize the distribution of RMT values according to parity and PPH history. Boxplots were constructed directly from the observed data and are presented using the median, interquartile range (IQR), and minimum–maximum values.

Given the exploratory nature of the study and the relatively small sample size, analyses should be interpreted as hypothesis-generating. Findings with borderline statistical significance were interpreted cautiously. All statistical tests were two-sided, and a *p*-value < 0.05 was considered statistically significant. Because the study was exploratory and hypothesis-generating in nature, formal adjustment for multiple testing was not applied. Accordingly, findings derived from secondary analyses and results with borderline statistical significance should be interpreted cautiously and considered exploratory pending confirmation in larger prospective studies. Variables included in the multivariable regression model were selected based on their clinical relevance to cesarean scar healing and/or their association with RMT in univariable analyses. Given the exploratory nature of the study and the limited sample size, the regression model was intended to identify potential independent associations rather than establish causal relationships.

## 3. Results

### 3.1. Baseline Demographic and Obstetric Characteristics

A total of 65 pregnant women with a history of at least one previous cesarean delivery were included in the final analysis. According to first-trimester RMT, 37 women were classified into the Thin Scar Group (RMT ≤ 8 mm) and 28 women into the Control Group (RMT > 8 mm).

Baseline demographic and obstetric characteristics are summarized in Table 1. Maternal age was comparable between groups, with a mean age of 31.57 ± 4.71 years in the Thin Scar Group and 30.29 ± 4.20 years in the Control Group (*p* = 0.260). Similarly, the interval since the first CS, gestational age at the first cesarean delivery, and interval since the second CS did not differ significantly between groups (all *p* > 0.05).

Significant differences were observed regarding parity and the number of previous cesarean deliveries. Women in the Thin Scar Group were more likely to have a parity of two or greater compared with controls (56.8% vs. 25.0%, *p* = 0.013). Likewise, multiple previous cesarean deliveries were significantly more common among women with thinner residual myometrium. While 89.3% of women in the Control Group had undergone only one previous CS, this proportion was 56.8% in the Thin Scar Group. Conversely, two previous cesarean deliveries were observed in 35.1% of women in the Thin Scar Group compared with only 7.1% of controls (*p* = 0.016).

No significant difference was observed regarding the mode of the first cesarean delivery. Emergency CS accounted for 51.4% of cases in the Thin Scar Group and 60.7% in the Control Group (*p* = 0.615).

Intraobserver reproducibility analysis demonstrated excellent agreement for RMT measurements (ICC = 0.92, 95% CI: 0.86–0.96), indicating high measurement reliability.

### 3.2. Pregnancy Characteristics and Ultrasonographic Assessment

Ultrasonographic findings and pregnancy outcomes are presented in Table 2. As expected, RMT differed markedly between study groups. The mean RMT was 5.48 ± 1.53 mm in the Thin Scar Group and 10.40 ± 1.85 mm in the Control Group (*p* < 0.001).

Mean cervical length did not differ significantly between groups (40.78 ± 5.86 mm vs. 38.96 ± 6.48 mm, *p* = 0.241). Similarly, placental localization showed no significant association with scar thickness. Anterior, posterior, fundal, and lateral placental implantation sites were distributed similarly between groups (*p* = 0.661).

Pregnancy outcomes were also comparable. Mean gestational age at delivery was 37.97 ± 1.94 weeks in the Thin Scar Group and 37.64 ± 1.85 weeks in the Control Group (*p* = 0.490). No statistically significant differences were observed regarding neonatal condition at birth. Mean 1 min Apgar scores were 7.89 ± 1.63 and 8.39 ± 1.96, respectively (*p* = 0.153), while mean 5 min Apgar scores were 8.95 ± 1.65 and 9.36 ± 0.56 (*p* = 0.211).

Likewise, neonatal birth weight was similar between groups, with mean values of 3138.65 ± 465.96 g and 3161.79 ± 494.95 g in the Thin Scar and Control Groups, respectively (*p* = 0.848).

### 3.3. Correlation Between RMT and Clinical Variables

Correlation analysis was performed to investigate associations between RMT and clinical characteristics (Table 3).

Among all evaluated variables, the number of previous cesarean deliveries demonstrated the strongest association with RMT. A moderate inverse correlation was identified between previous CS number and RMT (r = −0.463, *p* < 0.001), indicating progressive thinning of the residual myometrium with increasing numbers of cesarean deliveries.

No significant correlations were found between RMT and maternal age (r = −0.149, *p* = 0.237), interval since the first CS (r = −0.218, *p* = 0.081), cervical length (r = 0.034, *p* = 0.788), 5 min Apgar score (r = 0.226, *p* = 0.070), or neonatal birth weight (r = 0.240, *p* = 0.055). A weak positive correlation was observed between RMT and the 1 min Apgar score (r = 0.252, *p* = 0.043); however, given the modest correlation coefficient and the absence of significant associations with other neonatal outcomes, this finding should be interpreted cautiously.

**Figure 3:**
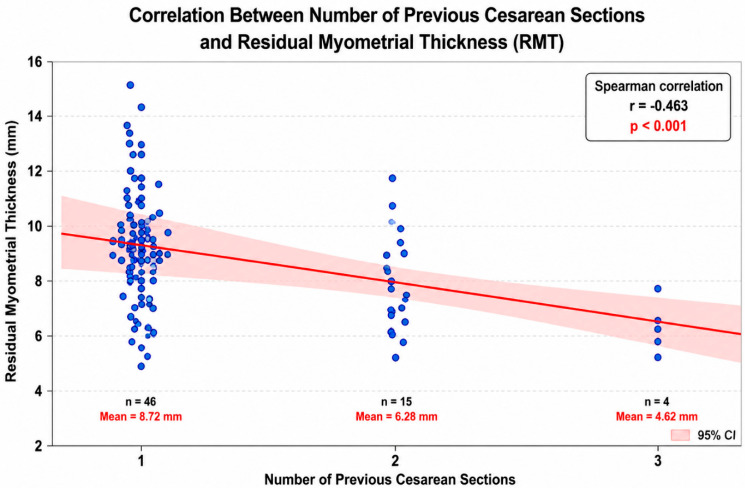
Correlation Between the Number of Previous Cesarean Deliveries and RMT. Scatter plot demonstrating the association between the number of previous cesarean deliveries and first-trimester RMT. Each point represents an individual patient. A moderate negative correlation was observed between increasing CS number and RMT (r = −0.463, *p* < 0.001), indicating that women with a greater number of previous cesarean deliveries tended to have thinner residual myometrium at the cesarean scar site. The solid line represents the linear regression trend, and the shaded area indicates the 95% CI.

The relationship between the number of previous cesarean deliveries and RMT is illustrated in Figure 3, which demonstrates a clear downward trend in scar thickness with increasing CS number.

### 3.4. Multivariable Linear Regression Analysis

To identify independent factors associated with RMT, a multivariable linear regression model was constructed including maternal age, parity, number of previous cesarean deliveries, interval since the previous CS, history of PPH, and mode of previous cesarean delivery (Table 4).

After adjustment for potential confounding factors, the number of previous cesarean deliveries remained the only variable independently associated with reduced RMT (β = −1.68, 95% CI: −2.84 to −0.52, *p* = 0.006). Specifically, each additional previous cesarean delivery was associated with an estimated decrease of approximately 1.7 mm in first-trimester RMT.

In contrast, maternal age (*p* = 0.42), parity (*p* = 0.19), interval since the previous CS (*p* = 0.24), history of PPH (*p* = 0.081), and mode of previous cesarean delivery (*p* = 0.65) were not independently associated with RMT after adjustment. Although PPH demonstrated a significant association with reduced RMT in univariable analyses, this relationship did not remain statistically significant in the multivariable model.

The final regression model explained approximately 28% of the variability in RMT (adjusted R^2^ = 0.28, model *p* = 0.002), indicating that previous CS number was the strongest determinant of first-trimester cesarean scar thickness in the study population.

### 3.5. RMT According to Obstetric Characteristics

The relationship between RMT and selected obstetric characteristics is shown in Table 5.

**Figure 4:**
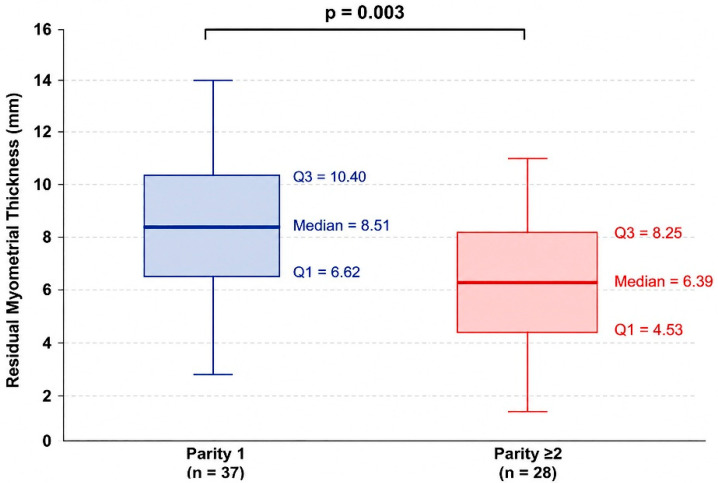
RMT according to parity. Women with parity ≥ 2 demonstrated significantly lower RMT than women with parity 1 (6.39 ± 2.76 mm vs 8.51 ± 2.81 mm, respectively; *p* = 0.003). The central horizontal line represents the median, whereas the lower and upper boundaries of the box represent the first (Q1) and third (Q3) quartiles, respectively. Whiskers indicate the minimum and maximum observed values. These findings suggest an association between increasing parity and reduced RMT at the site of the previous cesarean scar.

Mean RMT decreased progressively with increasing parity. Women with parity 1 had a mean RMT of 8.51 ± 2.81 mm, whereas women with parity ≥ 2 demonstrated a significantly lower mean RMT of 6.39 ± 2.76 mm (*p* = 0.003). This distribution is presented in Figure 4. The median RMT was 8.51 mm (Q1–Q3: 6.62–10.40 mm) among women with parity 1 and 6.39 mm (Q1–Q3: 4.53–8.25 mm) among those with parity ≥ 2.

**Figure 5:**
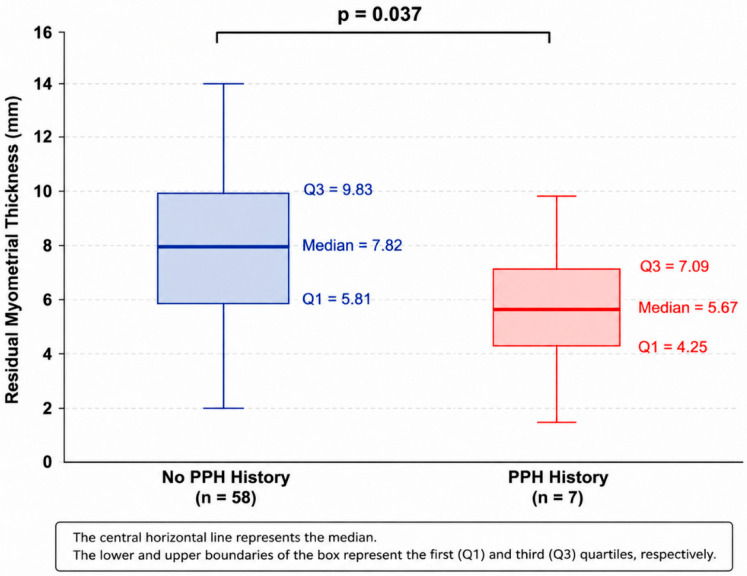
RMT according to PPH history. Women with a previous history of PPH demonstrated significantly lower RMT than those without such a history (5.67 ± 2.11 mm vs 7.82 ± 2.98 mm, respectively; *p* = 0.037). The central horizontal line represents the median, whereas the lower and upper boundaries of the box represent the first (Q1) and third (Q3) quartiles, respectively. Whiskers indicate the minimum and maximum observed values. These findings suggest an association between a history of PPH and reduced RMT at the site of the previous cesarean scar.

RMT also differed according to PPH history. Women without a history of PPH had a mean RMT of 7.82 ± 2.98 mm, whereas those with previous PPH demonstrated a significantly lower mean RMT of 5.67 ± 2.11 mm (*p* = 0.037). Figure 5 illustrates the corresponding distribution of RMT values. Median RMT values were 7.82 mm (Q1–Q3: 5.81–9.83 mm) and 5.67 mm (Q1–Q3: 4.25–7.09 mm), respectively.

No statistically significant associations were observed between RMT and gravidity (*p* = 0.199), abortion history (*p* = 0.771), mode of first cesarean delivery (*p* = 0.772), placental location (*p* = 0.657), or antenatal bleeding (*p* = 0.665).

## 4. Discussion

In this retrospective cohort study, we evaluated first-trimester RMT in women with a previous cesarean delivery and investigated its relationship with demographic, obstetric, and pregnancy-related characteristics. The principal findings of the present study were threefold. First, increasing numbers of previous cesarean deliveries were significantly associated with reduced first-trimester RMT. Second, women with higher parity demonstrated significantly thinner residual myometrium at the cesarean scar site. Third, a history of PPH was associated with lower RMT measurements. In contrast, maternal age, interpregnancy interval, gestational age at previous cesarean delivery, cervical length, placental location, gestational age at delivery, and neonatal birth weight were not significantly associated with scar thickness. Collectively, these findings support the hypothesis that cumulative uterine surgical exposure and reproductive history may have a greater influence on cesarean scar morphology than characteristics of the ongoing pregnancy itself. Furthermore, our findings reinforce the concept that first-trimester transvaginal sonographic assessment of cesarean scar integrity may provide valuable information regarding long-term uterine remodeling and healing following cesarean delivery [16,17,18,19].

The strongest association identified in our cohort was between the number of previous cesarean deliveries and RMT. Women with thinner scars were significantly more likely to have undergone multiple previous cesarean deliveries, and correlation analysis demonstrated a moderate inverse relationship between cesarean number and RMT. These findings are consistent with accumulating evidence suggesting that repeated cesarean incisions may progressively impair myometrial remodeling and scar healing. Vervoort et al. proposed that repeated disruption of the lower uterine segment, together with local inflammatory and healing responses, may contribute to niche formation and residual myometrial deficiency [17]. Earlier investigations evaluating lower uterine segment integrity have similarly demonstrated associations between abnormal scar morphology and factors related to previous cesarean delivery, including surgical characteristics and inter-delivery variables [20]. More recently, Wang et al. identified repeated cesarean delivery as one of the strongest predictors of large niche development and demonstrated an accumulation effect of multiple risk factors on scar deterioration [21]. Similarly, Zhou et al. reported that women with CSDs had significantly thinner residual muscular layers and that previous cesarean frequency was associated with impaired scar healing [22]. Histopathological evidence further supports this relationship. Tahermanesh et al. demonstrated a significant association between niche formation and increasing numbers of prior cesarean deliveries and confirmed that scar abnormalities may occur even after a single cesarean procedure [23]. Furthermore, current consensus statements continue to recognize previous cesarean delivery as the major determinant of subsequent niche formation and cesarean scar disorder [24]. Taken together, these findings support the concept that repeated cesarean delivery may progressively compromise myometrial integrity, resulting in measurable reductions in first-trimester RMT. However, the multivariable model explained only approximately 28% of the variability in RMT, indicating that a substantial proportion of scar-thickness variability remains unexplained and is likely influenced by additional biological, surgical, and obstetric factors not captured in the present study.

Parity also emerged as a significant determinant of scar thickness in the present study. Women with parity ≥ 2 exhibited substantially lower RMT values compared with women who had delivered only once. Although parity and previous cesarean number are closely related, increasing parity may represent cumulative exposure to uterine stretching, remodeling, and mechanical stress over successive pregnancies. The biological mechanisms underlying this association remain incompletely understood. However, previous studies have suggested that scar healing is influenced not only by surgical factors but also by individual wound-healing capacity, inflammatory responses, and repeated uterine remodeling processes [17,25]. The multifactorial model proposed by Wang et al. further supports the concept that scar integrity reflects the cumulative effect of multiple obstetric and patient-related factors rather than a single determinant [21]. Our findings therefore suggest that higher parity may contribute to progressive thinning of the residual myometrium and should be considered when evaluating scar morphology during subsequent pregnancies.

An additional finding of our study was the association between a previous history of PPH and reduced RMT. Although only a limited number of women reported prior PPH, those patients demonstrated significantly thinner residual myometrium than women without such a history. The exact mechanism underlying this relationship remains speculative. PPH may reflect more complex deliveries, increased tissue trauma, impaired uterine contractility, infection-related morbidity, or altered healing processes following cesarean delivery. Previous literature has highlighted the importance of myometrial integrity in the pathophysiology of abnormal placentation and hemorrhagic obstetric complications [26,27]. In addition, contemporary evidence increasingly supports the concept that deficient myometrial healing may represent a common biological substrate linking scar defects, abnormal placentation, and future reproductive complications [27,28]. While causality cannot be inferred from our findings, the observed association suggests that previous PPH may represent a marker of impaired uterine healing and warrants further investigation in larger prospective studies.

A weak positive correlation was observed between RMT and the 1 min Apgar score; however, this finding should be interpreted cautiously given the modest correlation coefficient, the absence of significant associations with other neonatal outcomes, and the exploratory nature of the analyses. No significant relationship was identified between RMT and 5 min Apgar score, gestational age at delivery, or neonatal birth weight. Collectively, these findings suggest that reduced first-trimester RMT is unlikely to have a major influence on short-term neonatal outcomes in otherwise uncomplicated pregnancies. This observation is consistent with current understanding that scar abnormalities primarily reflect maternal structural characteristics rather than determinants of immediate neonatal well-being. Accordingly, several studies evaluating niche morphology have emphasized gynecological symptoms, reproductive consequences, and future obstetric risks rather than neonatal outcomes [16,29,30].

The absence of significant associations between RMT and maternal age, interval since previous cesarean delivery, cervical length, placental location, abortion history, or antenatal vaginal bleeding also deserves consideration. Previous studies have reported conflicting findings regarding the influence of these factors on scar healing. While some investigators have suggested that interpregnancy interval and maternal characteristics may contribute to scar remodeling, others have failed to demonstrate consistent effects [18,20,22]. The lack of significant associations in our cohort may indicate that these variables exert weaker effects than cumulative surgical exposure. Alternatively, the relatively modest sample size may have limited the statistical power to detect small differences.

An important aspect of the present study is the use of first-trimester TVUS for scar assessment. Growing evidence supports the feasibility and reproducibility of early pregnancy scar evaluation. Stirnemann et al. demonstrated that cesarean scars can be reliably identified during first-trimester ultrasound examinations [29], while Baranov et al. reported strong agreement between scar characteristics assessed before pregnancy and at 11–14 weeks of gestation [31]. More recent investigations have further confirmed that transvaginal ultrasound provides reproducible measurements of scar morphology and RMT during pregnancy [18,19,32]. The increasing recognition of CSDs and cesarean scar disorder has reinforced the importance of standardized sonographic assessment and terminology [24,33]. Furthermore, recent evidence has suggested that niche characteristics identified by ultrasonography may have implications for future obstetric outcomes, including abnormal placentation, cesarean scar pregnancy, and uterine rupture, although the available evidence remains heterogeneous and further prospective studies are needed [28,34]. Our findings support the growing body of evidence suggesting that first-trimester RMT measurement may serve as a practical marker of scar integrity in women with previous cesarean delivery. It should be noted that RMT represents only one aspect of cesarean scar morphology. Contemporary international recommendations advocate comprehensive niche characterization incorporating niche dimensions and adjacent myometrial measurements. Therefore, while RMT provides a practical and reproducible marker of scar integrity, it may not fully capture the complexity of cesarean scar morphology.

The potential clinical implications of reduced RMT extend beyond the current pregnancy, although the predictive value of first-trimester RMT for future obstetric outcomes remains incompletely established. Increasing evidence indicates that scar defects and residual myometrial deficiency may be associated with abnormal uterine bleeding, pelvic pain, infertility, cesarean scar pregnancy, placenta accreta spectrum disorders, and other reproductive complications [16,28,30,31]. Recent consensus recommendations have further emphasized that management decisions should incorporate RMT, symptom burden, and future fertility plans [24]. Nevertheless, contemporary consensus statements also emphasize that sonographic scar abnormalities should not automatically be equated with clinical disease and that asymptomatic patients do not necessarily require intervention [24,33]. In this context, RMT should be interpreted primarily as a marker of scar morphology rather than as an isolated predictor of adverse outcomes. Accordingly, the present findings should be interpreted as demonstrating sonographic associations rather than clinically validated predictive relationships. Although routine clinical decision-making should not rely solely on first-trimester RMT measurements, early identification of women with relatively thin residual myometrium may contribute to individualized counseling and may help inform future surveillance strategies in conjunction with clinical history, symptom profile, and other sonographic findings. The present study contributes to this evolving field by identifying clinical factors associated with reduced scar thickness during early pregnancy.

Recent evidence also suggests that surgical factors may substantially influence long-term scar healing. Randomized data indicate that uterine closure technique can affect subsequent niche formation and RMT [35]. In addition, a systematic review and meta-analysis by Qayum et al. demonstrated greater residual myometrial thickness following double-layer uterine closure compared with single-layer closure, whereas a more recent meta-analysis by Dominoni et al. reported lower rates of uterine scar niche formation after single-layer closure. These findings suggest that uterine closure strategy may influence different aspects of scar healing and morphology, including both residual myometrial thickness and niche development [36,37]. While emerging studies evaluating barbed sutures and myometrial reconstruction have reported favorable scar characteristics following optimized surgical repair [25,27], contemporary reviews have further proposed that preservation of tissue perfusion, adequate myometrial approximation, and balanced tension distribution during uterine closure may play important roles in long-term scar remodeling [25]. Unfortunately, detailed information regarding uterine closure methods and suture materials used during previous cesarean deliveries was unavailable in our cohort. Therefore, the potential contribution of surgical technique to the observed differences in RMT could not be evaluated. Consequently, the observed associations identified in the present study should not be interpreted as causal relationships independent of surgical factors.

The strengths of the present study include the use of first-trimester ultrasound measurements obtained using a standardized transvaginal sonographic protocol and the assessment of clinically relevant obstetric outcomes in a well-defined cohort of women with previous cesarean delivery. Furthermore, all measurements were obtained using a consistent methodology under expert supervision, reducing potential measurement variability. The study additionally contributes data from a period of pregnancy that remains relatively underrepresented in the literature, namely the first trimester, when scar morphology can be evaluated before substantial lower uterine segment remodeling occurs. By focusing on early-pregnancy RMT and its associations with maternal, obstetric, and pregnancy-related characteristics, the present study provides additional insight into factors associated with cesarean scar morphology at a stage when standardized scar assessment may be less influenced by the anatomical changes that occur later in gestation.

**Figure 6:**
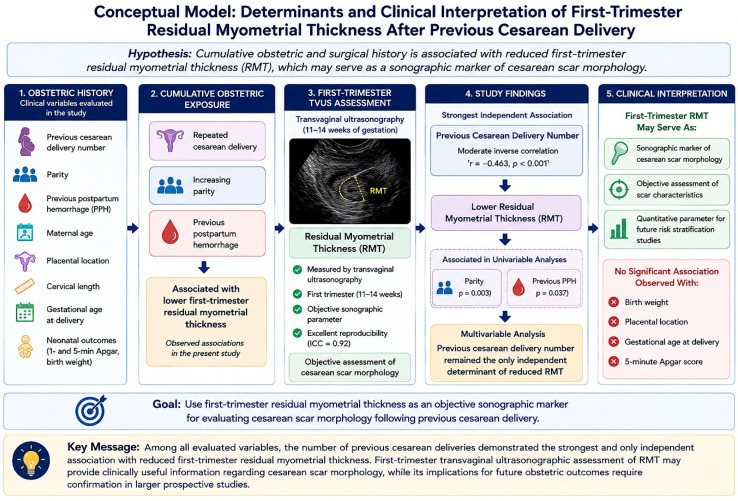
Conceptual model summarizing the determinants and clinical interpretation of first-trimester RMT following previous cesarean delivery. The model illustrates the observed associations between cumulative obstetric and surgical history and first-trimester RMT measured by TVUS. Previous cesarean delivery number demonstrated the strongest association with reduced RMT among the variables evaluated and was the only factor that retained statistical significance after multivariable adjustment, although additional determinants of scar morphology likely contribute to the variability in RMT. The figure also highlights variables that were not significantly associated with RMT in the present study and summarizes the potential role of first-trimester RMT as an objective sonographic marker of cesarean scar morphology.

To facilitate interpretation of the present findings, a conceptual model summarizing the determinants and potential clinical implications of first-trimester RMT is presented in Figure 6. The model illustrates how cumulative obstetric and surgical history, particularly increasing numbers of previous cesarean deliveries, may be associated with reduced RMT measured during early pregnancy. In the current cohort, the number of previous cesarean deliveries demonstrated the strongest association with lower RMT among the variables evaluated and remained the only factor that retained statistical significance after multivariable adjustment, whereas parity and a history of PPH were associated with RMT only in univariable analyses. Nevertheless, the multivariable model explained only a modest proportion of RMT variability, suggesting that additional determinants of scar morphology remain to be identified. The model further highlights the role of TVUS as an objective and reproducible method for assessing RMT and characterizing cesarean scar morphology during the first trimester. Importantly, several maternal, sonographic, and neonatal variables, including placental location, gestational age at delivery, birth weight, and 5 min Apgar score, were not significantly associated with RMT in the present study. Therefore, the framework presented in Figure 6 should be interpreted as a clinical and sonographic representation of the observed associations rather than a mechanistic model of scar healing. Collectively, these findings support the concept that first-trimester RMT may reflect cumulative obstetric and surgical exposure and may serve as a useful sonographic parameter for future investigations of cesarean scar characteristics and their potential clinical relevance.

Several limitations should be acknowledged. First, this was a single-center study with a relatively small sample size, which may limit the generalizability of the findings and reduce statistical power for detecting weaker associations. Second, the observational design precludes causal inference regarding the relationship between clinical variables and scar thickness. Third, the present analysis focused primarily on RMT, which was the only scar-related parameter consistently available across the retrospective dataset. Standardized niche-characterization variables, including niche morphology, niche volume, residual-to-adjacent myometrial thickness ratio, and niche classification, were not consistently recorded and therefore could not be evaluated. Fourth, information regarding several established determinants of cesarean scar healing was unavailable because of the retrospective design of the study. These variables included uterine position, uterine retroflexion, labor characteristics before previous cesarean delivery, cervical dilatation, postoperative infection, endometritis, body mass index, smoking status, metabolic comorbidities, and detailed surgical closure techniques. As these factors have been reported to influence scar morphology and healing, residual confounding cannot be excluded and may have contributed to the unexplained variability in RMT measurements [17,20,21,22,35]. In addition, parity and the number of previous cesarean deliveries are biologically and clinically related variables. Although both factors were included in the multivariable model because of their potential relevance to scar healing, residual interrelationships between these variables may have influenced the stability of the regression estimates. Therefore, the multivariable findings should be interpreted cautiously and require confirmation in larger prospective cohorts. Fifth, symptoms related to cesarean scar disorder, including abnormal uterine bleeding, pelvic pain, dyspareunia, and infertility, were not systematically assessed [24,29,30,33]. Consequently, correlations between sonographic findings and clinical symptomatology could not be investigated. Sixth, serial ultrasound assessments throughout pregnancy were not performed, preventing evaluation of dynamic changes in scar morphology over time [18,31,32]. Seventh, pathological confirmation of sonographic findings was not available, and therefore structural scar characteristics could not be correlated with histological healing patterns [23]. Eighth, participants were categorized using an exploratory 8 mm RMT threshold for comparative analyses. Although this approach facilitated clinically interpretable group comparisons, dichotomization of a continuous variable may result in information loss and potential misclassification. To mitigate this limitation, the principal findings were additionally evaluated using continuous-variable correlation and multivariable regression analyses. Ninth, multiple exploratory comparisons and correlation analyses were performed without formal adjustment for multiple testing. Therefore, findings with borderline statistical significance, particularly those observed in secondary analyses, should be interpreted cautiously and require confirmation in larger prospective studies. Tenth, although all ultrasound examinations were performed during the first-trimester screening period (11–14 weeks of gestation) according to the institutional protocol, the exact gestational age at the time of individual RMT measurements was not consistently available in the retrospective dataset. Consequently, potential variation in RMT according to gestational age within this limited first-trimester interval could not be evaluated or incorporated into the regression analyses. Future prospective studies should record gestational age at the time of scar assessment to determine whether temporal variation within the first trimester influences RMT measurements. Finally, because the study was not designed to investigate placenta accreta spectrum, cesarean scar pregnancy, uterine rupture, fertility outcomes, or long-term reproductive sequelae, the clinical implications of reduced RMT beyond the index pregnancy remain uncertain [16,26,28,29].

## 5. Conclusions

In conclusion, first-trimester RMT was significantly associated with the number of previous cesarean deliveries, parity, and a history of PPH. Among the evaluated variables, the number of previous cesarean deliveries demonstrated the strongest association with reduced scar thickness and was the only factor that retained statistical significance after multivariable adjustment. Increasing cesarean number was associated with progressively lower RMT, suggesting cumulative effects of repeated uterine surgery on cesarean scar morphology. However, the multivariable model explained only a modest proportion of RMT variability, indicating that additional biological, surgical, and obstetric determinants of scar morphology remain incompletely understood. In contrast, most maternal, sonographic, and neonatal characteristics were not significantly related to RMT. These findings suggest that first-trimester transvaginal assessment of RMT may serve as a useful sonographic marker of cesarean scar morphology and healing characteristics in women with previous cesarean delivery. Larger multicenter prospective studies incorporating standardized niche assessment, serial ultrasound evaluation throughout pregnancy, symptom evaluation, surgical variables, and long-term obstetric and reproductive outcomes together with clinically relevant maternal outcomes are required to further clarify the clinical significance of reduced RMT.

## Figures and Tables

**Table 1:** Baseline Demographic and Obstetric Characteristics of the Study Population.

Variable	Total	Study	Control	*p*
Age (years)	31.02 ± 4.51	31.57 ± 4.71	30.29 ± 4.20	0.26
Parity = 1	37 (56.9%)	16 (43.2%)	21 (75.0%)	0.013
Parity ≥ 2	28 (43.1%)	21 (56.8%)	7 (25.0%)	
Previous CS = 1	46 (70.8%)	21 (56.8%)	25 (89.3%)	0.016
Previous CS = 2	15 (23.1%)	13 (35.1%)	2 (7.1%)	
Previous CS = 3	4 (6.2%)	3 (8.1%)	1 (3.6%)	
Time since 1st CS (years)	6.09 ± 4.00	6.62 ± 4.40	5.39 ± 3.37	0.224
1st CS Emergency	36 (55.4%)	19 (51.4%)	17 (60.7%)	0.615
1st CS Elective	29 (44.6%)	18 (48.6%)	11 (39.3%)	
Gestational week of 1st CS	37.62 ± 1.93	37.49 ± 1.94	37.79 ± 1.95	0.541
Time since 2nd CS (years)	5.53 ± 3.67	5.81 ± 3.90	4.00 ± 1.73	0.449

**Table 2:** Ultrasonographic Findings and Pregnancy Outcomes.

Variable	Total (n = 65)	Study (n = 37)	Control (n = 28)	*p*
Myometrium thickness (mm)	7.60 ± 2.97	5.48 ± 1.53	10.40 ± 1.85	<0.001
Cervical length (mm)	40.00 ± 6.16	40.78 ± 5.86	38.96 ± 6.48	0.241
Placenta anterior	17 (26.2%)	11 (29.7%)	6 (21.4%)	
Placenta posterior	19 (29.2%)	11 (29.7%)	8 (28.6%)	0.661
Placenta fundal	17 (26.2%)	10 (27.0%)	7 (25.0%)	
Placenta lateral	12 (18.5%)	5 (13.5%)	7 (25.0%)	
Gestational age at delivery (week)	37.83 ± 1.89	37.97 ± 1.94	37.64 ± 1.85	0.490
APGAR 1 min	8.11 ± 1.39	7.89 ± 1.63	8.39 ± 1.96	0.153
APGAR 5 min	9.12 ± 1.31	8.95 ± 1.65	9.36 ± 0.56	0.211
Birth weight (g)	3148.62 ± 474.99	3138.65 ± 465.96	3161.79 ± 494.95	0.848

**Table 3:** Correlation Analysis Between RMT and Clinical Variables.

Variable	r	*p*
Age	−0.149	0.237
Previous CS number	−0.463	<0.001
Time since 1st CS (years)	−0.218	0.081
Gestational week of 1st CS	0.171	0.173
Time since 2nd CS (years)	−0.401	0.089
Gestational week of 2nd CS	0.412	0.112
Cervical length	0.034	0.788
APGAR 1 min	0.252	0.043
APGAR 5 min	0.226	0.070
Birth weight	0.240	0.055

**Table 4:** Multivariable Linear Regression Analysis for Factors Associated with RMT.

Variable	β Coefficient	Standard Error	95% CI	*p*
Maternal age (years)	−0.04	0.05	−0.14 to 0.06	0.42
Previous CS number	−1.68	0.58	−2.84 to −0.52	0.006
Parity ≥ 2	−0.61	0.47	−1.55 to 0.33	0.19
Time since previous CS (years)	−0.07	0.06	−0.19 to 0.05	0.24
Previous postpartum hemorrhage	−1.29	0.73	−2.75 to 0.17	0.081
Emergency vs elective CS	0.22	0.49	−0.76 to 1.20	0.65

**Table 5:** Differences in RMT According to Various Parameters.

Variable	n	Mean RMT	SD	*p*
Gravida				0.199
2	23	8.39	2.42	
3	27	7.45	3.27	
≥4	15	6.65	3.02	
Parity				0.003
1	37	8.51	2.81	
≥2	28	6.39	2.76	
Abortus				0.771
None	41	7.51	2.79	
≥1	24	7.74	3.30	
1st CS type				0.772
Emergency	36	7.69	3.07	
Elective	29	7.47	2.48	
Placental location				0.657
Anterior	17	7.50	3.22	
Posterior	19	7.64	3.29	
Fundal	17	7.03	2.90	
Lateral	12	8.46	2.16	
PPH history				0.037
No	58	7.82	2.98	
Yes	7	5.67	2.11	
Antenatal vaginal bleeding				0.665
No	63	7.57	3.01	
Yes	2	8.50	0.71	

## Data Availability

The datasets generated and/or analyzed during the current study are available from the corresponding author upon reasonable request.

## Abbreviations

ANOVA	Analysis of Variance
CI	Confidence Interval
CS	Cesarean Section
CSD	Cesarean Scar Defect
ICC	Intraclass Correlation Coefficient
PPH	Postpartum Hemorrhage
RMT	Residual Myometrial Thickness
TVUS	Transvaginal Ultrasonography
